# Gut Metabolite TMAO Induces Synaptic Plasticity Deficits by Promoting Endoplasmic Reticulum Stress

**DOI:** 10.3389/fnmol.2020.00138

**Published:** 2020-08-12

**Authors:** Manoj Govindarajulu, Priyanka D. Pinky, Ian Steinke, Jenna Bloemer, Sindhu Ramesh, Thiruchelvan Kariharan, Robert T. Rella, Subhrajit Bhattacharya, Muralikrishnan Dhanasekaran, Vishnu Suppiramaniam, Rajesh H. Amin

**Affiliations:** ^1^Department of Drug Discovery and Development, Harrison School of Pharmacy, Auburn University, Auburn, AL, United States; ^2^Center for Neuroscience, Auburn University, Auburn, AL, United States; ^3^Department of Pharmaceutical and Biomedical Sciences, Touro College of Pharmacy, New York, NY, United States

**Keywords:** trimethylamine N-oxide (TMAO), synaptic plasticity, long-term potentiation (LTP), endoplasmic reticulum stress (ER stress), insulin resistance, Alzheimer’s disease, dysbiosis

## Abstract

Dysbiosis of gut microbiota is strongly associated with metabolic diseases including diabetes mellitus, obesity, and cardiovascular disease. Recent studies indicate that Trimethylamine N-oxide (TMAO), a gut microbe-dependent metabolite is implicated in the development of age-related cognitive decline. However, the mechanisms of the impact of TMAO on neuronal function has not been elucidated. In the current study, we investigated the relationship between TMAO and deficits in synaptic plasticity in an Alzheimer’s model (3×Tg-AD) and insulin resistance (Leptin deficient db/db) mouse by measuring plasma and brain levels of TMAO. We observed increased TMAO levels in the plasma and brain of both db/db and 3×Tg-AD mice in comparison to wild-type mice. Besides, TMAO levels further increased as mice progressed in age. Deficits in synaptic plasticity, in the form of reduced long-term potentiation (LTP), were noted in both groups of mice in comparison to wild-type mice. To further explore the impact of TMAO on neuronal function, we utilized an *ex-vivo* model by incubating wild-type hippocampal brain slices with TMAO and found impaired synaptic transmission. We observed that TMAO induced the PERK-EIF2α-ER stress signaling axis in TMAO treated *ex-vivo* slices as well as in both db/db and 3×Tg-AD mice. Lastly, we also observed altered presynaptic and reduced postsynaptic receptor expression. Our findings suggest that TMAO may induce deficits in synaptic plasticity through the ER stress-mediated PERK signaling pathway. Our results offer novel insight into the mechanism by which TMAO may induce cognitive deficits by promoting ER stress and identifies potential targets for therapeutic intervention.

## Introduction

Recent investigation for understanding the mechanism by which altered gut-brain axis impacts the central nervous system has received significant attention. The alterations in the gut microbiome termed dysbiosis has been observed to be associated with neurological conditions including dementia, autism, multiple sclerosis, and Parkinson’s disease (Fung et al., 2017; Fung, 2020). In cognitively impaired elderly patients and in transgenic rodent models of Alzheimer’s disease (AD), gut dysbiosis had significantly positive correlations with AD pathology including cerebral amyloid-β depositions (Cattaneo et al., 2017). Trimethylamine N-oxide (TMAO), a hepatic metabolite, comes from the gut microbiota catabolism of dietary nutrients (primarily L-carnitine, choline, and phosphatidylcholine; Zhu et al., 2017). Interestingly, higher concentrations of TMAO in circulation and cerebral spinal fluid (CSF) have been observed in diabetic patients as well as patients with mild cognitive impairment (MCI) and AD dementia (Vogt et al., 2018). Recent findings have reported that TMAO promotes brain aging and cognitive impairment (Li et al., 2018).

However, the impact of TMAO on neuronal function and signaling mechanisms associated with altered synaptic plasticity observed in patients with memory impairment and AD is unknown. Importantly, TMAO coordinates the progression of cardiovascular diseases and metabolic disorders, by inducing the Endoplasmic Reticulum (ER) stress signaling pathway by binding to the ER stress protein PERK (Chen et al., 2019). ER stress has been observed to modulate the deposition of misfolded amyloid peptides in the brain and subsequently affects cognitive processes (Halliday and Mallucci, 2015). It has been suggested that activation of PERK may be responsible for cognitive deficiencies in AD (Duran-Aniotz et al., 2014) by altering active protein synthesis through modulation of eIF2α phosphorylation (Costa-Mattioli et al., 2005, 2009; Jiang et al., 2010). Furthermore, PERK appears to influence hippocampal synaptic plasticity, as PERK deficient mice displayed enhanced mGluR mediated long-term depression (Trinh et al., 2014). Additionally, the inhibition of hippocampal PERK in the CA1 region of the hippocampus enhanced neuronal excitability and cognitive function (Sharma et al., 2018).

In the current study, we investigated the impact of the gut-brain signaling axis on hippocampal synaptic plasticity. We tested the hypothesis that elevated TMAO levels observed in models of cognitive impairment (3×Tg-AD and db/db) mice are associated with impaired long-term potentiation (LTP) and synaptic plasticity. Second, we determined that hippocampal exposure to TMAO induces ER stress involving PERK and results in reduced synaptic plasticity.

## Materials and Methods

### Animals

3×Tg-AD mice [B6;129-Tg(APPSwe, tauP301L)] and control (C57BL/6) female mice (wild-type) were obtained from The Jackson Laboratory (stock #34830 and #101045, respectively). Db/db mice aged 8 months were purchased from Envigo (stock #17308M) were utilized for the study. According to the Jackson Laboratory, the 3×Tg-AD male mice do not exhibit phenotypic traits compared to females and hence we utilized female mice only. Similarly, we utilized female mice for db/db model to maintain uniformity and minimize gender variations. All mice had free access to food and water, group-housed in a temperature and humidity-controlled colony room with a 12:12 light/dark cycle (lights on at 6 a.m.). All experiments and procedures were conducted following the National Institute of Health (NIH) guidelines and approved by Auburn University Institutional Animal Care and Use Committee (IACUC).

### Chemicals

For hippocampal slice experiments, Trimethylamine N-oxide Dihydrate (TMAO) was purchased from Tokyo Chemical Industry. On the day of the experiment, TMAO was reconstituted in dimethyl sulfoxide (DMSO) at a stock concentration of 50 mM. The TMAO stock solution was diluted in the artificial CSF (ACSF) to a final concentration of 50 μM. All other chemicals were obtained from Millipore Sigma unless otherwise specified.

### TMAO Elisa Kit

Mouse TMAO Elisa was purchased from Abbkine (cat# KTE71902). Briefly Mouse TMAO ELISA Kit is based upon a two-site sandwich ELISA to quantitate TMAO in samples. Standards and samples are based upon known concentrations of TMAO present which is bound by the immobilized antibody. Evaluation of TMAO concentrations is based upon a colorimetric assay where the color intensity is measured at an absorbance of 450 nm. Concentrations of TMAO in samples are evaluated based upon a standard curve.

### TMAO Elisa

#### Serum

Serum samples from three independent mice per group; wild-type 8 months, wild-type 18 months, 3×Tg-AD 8 months, 3×Tg-AD 18 months, and leptin receptor-deficient diabetic mice (db/db) 8 months were used to determine serum concentrations. Concentrations were determined by subtracting the background value and using the standard calibration curve equation.

#### For Brain Tissue

Brain tissue (hippocampus and cortex) was weighed, minced, and homogenized in PBS on ice. Samples were then sonicated and centrifuged for 5 min at 5,000× *g*. The supernatant was collected and standardized for protein content. Fifty microliter from each sample from three independent mice per group discussed above, were run in triplicate. Absorbance was determined by standardizing to protein concentrations of samples. Concentrations were then calculated using the standard calibration curve equation.

### Oral Glucose Tolerance Test

Control (wild-type) mice, 3×Tg-AD, and db/db mice fasted overnight and glucose was administered intraperitoneal with glucose (2 g/kg). Following glucose administration, blood samples were collected from the tail vein at 0, 30, 60, 90, and 120 min. The contents of blood glucose were evaluated by using a Contour Next glucometer and glucose strips.

### Preparations of Acute Hippocampal Slices

All animals were housed in a vivarium maintained on a 12 h:12 h light: dark cycle and at a temperature of 24°C. C57BL/6 mice at 8 months were euthanized with carbon dioxide (CO_2_) and decapitated to remove the brain. The brain was then washed with the ice-cold oxygenated cutting solution (NaCl 85 mM, KCl 2.5 mM, MgSO_4_ 4.0 mM, CaCl_2_ 0.5 mM, NaH_2_PO_4_ 1.25 mM, NaHCO_3_ 25 mM, glucose 25 mM, sucrose 75 mM, and ascorbate 0.5 mM) and sliced using a Leica VT1200S Vibratome (Leica Biosystems Inc., Buffalo Grove, IL, USA; 350 μm thick). The slices were preserved in a holding chamber, submerged in the oxygenated artificial CSF (aCSF, in mM 124 NaCl, 3 KCl, 1.5 MgSO_4_7H_2_0, 1.2 NaH_2_PO_4_, 2.4 CaCl_2_, 25 NaHCO_3_, and 10 D-Glucose bubbled with 95% O_2_/5% CO_2_). Hippocampal slices were incubated in aCSF with 0.03% DMSO vehicle (control) or 50 μM of TMAO for 4–6 h at 30°C, before starting the electrophysiology recordings (Bloemer et al., 2019). Similarly, hippocampal slice preparations were performed on Control (wild-type) mice, 3×Tg-AD and db/db mice at 8 months to perform LTP experiments.

### Extracellular Field Potential Recording

After at least 4-h of incubation, slices were transferred into a recording chamber for electrophysiological measurements as previously described (13–16) with continuous ACSF perfusion at 34°C. A bipolar stimulating electrode (MicroProbes, Gaithersburg, MD, USA) was placed in the Schaffer collateral pathway. An extracellular recording pipette drawn with the PC-10 Dual-Stage Glass Micropipette Puller (Narishige, Amityville, NY, USA) and filled with ACSF (2–6 MΩ) was placed in the stratum radiatum of CA1 to record field excitatory postsynaptic potentials (fEPSPs). For paired-pulse facilitation (PPF), pairs of stimuli were separated by varying intervals. Ratios of fEPSP slopes from the second stimulus (fESPS2) to fEPSP slopes from the first stimulus (fESPS1) were calculated and plotted as a function of interstimulus intervals. Basal synaptic transmission, represented by input-output responses, was determined as the slope of fEPSPs and plotted as a function of fiber volley amplitude. For LTP experiments, stimulus intensity was set at 50% of the amplitude at which the initial population spike appeared. LTP was induced after at least 10 min of stable baseline recording using a Theta Burst Stimulation (TBS) protocol (10 bursts of stimuli, each of four pulses at 100 Hz, the interburst interval of 200 ms, and 20 s intervals between individual sweeps), and recording was continued for 60 min post-TBS (Parameshwaran et al., 2012; Bhattacharya et al., 2015; Kariharan et al., 2015; Bloemer et al., 2019). LTP was measured as an average of fEPSP slopes from 50 to 60 min after the end of induction. For the induction data analysis, sweep analysis was computed by normalizing the amplitude of the first fEPSP of sweeps 2–5 with the amplitude of the first fEPSP of the first sweep. The data were recorded online using the WinLTP software (University of Bristol, UK). Standard off-line analyses of the data were conducted using Prism software (GraphPad Prism version 8, San Diego, CA, USA).

### Western Blot

TMAO and vehicle-control treated slices post-incubation control (wild-type) mice, 3×Tg-AD, and db/db mice brains were lysed in N-PER containing a protease and phosphatase inhibitor cocktail (Halt Protease and Phosphatase Inhibitor Cocktail, Thermo Fisher Scientific, Waltham, MA, USA). The liver lysates from control (wild-type) mice, 3×Tg-AD, and db/db mice were lysed in cell lysis buffer with Halt Protease and Phosphatase Inhibitor cocktail). Total protein was estimated by bicinchoninic acid assay-BCA assay (Pierce BCA Protein Assay Kit, Thermo Fisher Scientific, Waltham, MA, USA) and then stored at −80°C until use. Samples were mixed thoroughly with 4× Laemmli buffer (Alfa Aesar) and were loaded into 8–16% SurePAGE precast gel (GenScript Biotech). The proteins were transferred to nitrocellulose membranes (Immobilon-p Millipore, Darmstadt, Germany) and blocked with 5% Bovine serum albumin in Tris-buffered saline, 0.1% v/v Tween 20 (TBST) for 1 h. Membranes were washed with TBST and incubated with primary antibodies listed in Table 1. Membranes were probed with secondary anti-rabbit or anti-mouse antibody (Cell Signaling Technology, Danvers, MA, USA 1:5,000) for 1 h. Membranes were imaged with Biorad imager. Finally, the densities of these bands were normalized to GAPDH/alpha-tubulin.

**Table 1:** Summary of antibodies and working conditions used in the experiments.

Antibodies	Species	Source	Catalog#	Dilution
**Primary antibodies**
ATF-4	Rabbit	Cell Signaling Technology	11815	1:1,000
CHOP	Mouse	Cell Signaling Technology	2895	1:1,000
CREB	Mouse	Cell Signaling Technology	4820	1:1,000
Phospho-CREB	Rabbit	Cell Signaling Technology	9198	1:1,000
eIF2α	Rabbit	Cell Signaling Technology	5324	1:1,000
Phospho-eIF2α (Ser51)	Rabbit	Cell Signaling Technology	3398	1:1,000
FMO3	Rabbit	Abcam	126711	1:1,000
GAPDH	Rabbit	Cell Signaling Technology	5174	1:1,000
GluA1	Rabbit	Cell Signaling Technology	13185	1:1,000
GluA2	Rabbit	Cell Signaling Technology	13607	1:1,000
GluN2A	Rabbit	Cell Signaling Technology	4205	1:1,000
GluN2B	Rabbit	Cell Signaling Technology	14544	1:1,000
PERK	Rabbit	Cell Signaling Technology	5683	1:1,000
phospho-PERK	Rabbit	Cell Signaling Technology	3179	1:1,000
PSD95	Rabbit	Cell Signaling Technology	3450	1:1,000
Synaptophysin	Rabbit	Cell Signaling Technology	36406	1:1,000
α-tubulin	Mouse	DSHB	12G10	1:1,000
vGLUT1	Rabbit	Cell Signaling Technology	12331	1:1,000
**Secondary antibodies**
Anti-mouse IgG	Horse	Cell Signaling Technology	7074P2	1:5,000
Anti-rabbit IgG	Goat	Cell Signaling Technology	7076P2	1:5,000

### Reactive Oxygen Species Generation

The vehicle-treated and TMAO treated brain slices were homogenized in 0.1 M phosphate buffer (pH 7.8), using a glass-Teflon homogenizer, followed by centrifugation at 10,000 *g* for 60 min at 4°C and the supernatant was collected. Protein quantification was determined using the Thermo Scientific Pierce 660 nm Protein Assay reagent kit (Pierce, Rockford, IL, USA). The generation of reactive oxygen species in both the groups was estimated spectrofluorometrically by measuring the conversion of non-fluorescent chloromethyl-DCF-DA (2′,7-dichlorofluorescein diacetate, DCF-DA) to fluorescent DCF using an excitation wavelength of 492 nm and an emission wavelength of 527 nm. Readings were taken by a Thermo Scientific Varioskan Flash Spectral Scanning Multimode Reader. Results were expressed as a percentage change from the control.

### Lipid Peroxidation

Lipid peroxide content in vehicle-treated and TMAO treated brain slices was determined calorimetrically by measuring the amount of thiobarbituric acid-reactive substances (TBARS) formed in the plate reader at 532 nm. The results were expressed as a percentage change from the control.

### Hydrogen Peroxide Generation

Hydrogen peroxide generation in the vehicle-treated and TMAO treated brain slices were measured fluorometrically at Ex/Em 535/587 nm.

### Nitrite Content

Nitrite content in the vehicle-treated and TMAO treated brain slices was measured using Griess reagent. An azo product formed was measured spectrophotometrically at 545 nm.

### Statistical Analysis

Statistical analysis was performed using Prism 5.04 software. All data were assessed by unpaired or paired *t*-tests, or two-tailed, one-way ANOVA, where appropriate. Tukey *post hoc* comparisons were used to compare groups when ANOVA indicated significance, except where expected effects were assessed with planned pairwise comparisons. Results were considered statistically significant when *p* < 0.05. All data are presented as means ± SEM.

## Results

### Elevated TMAO Levels in 3×Tg-AD and db/db Mice

**Figure 1:**
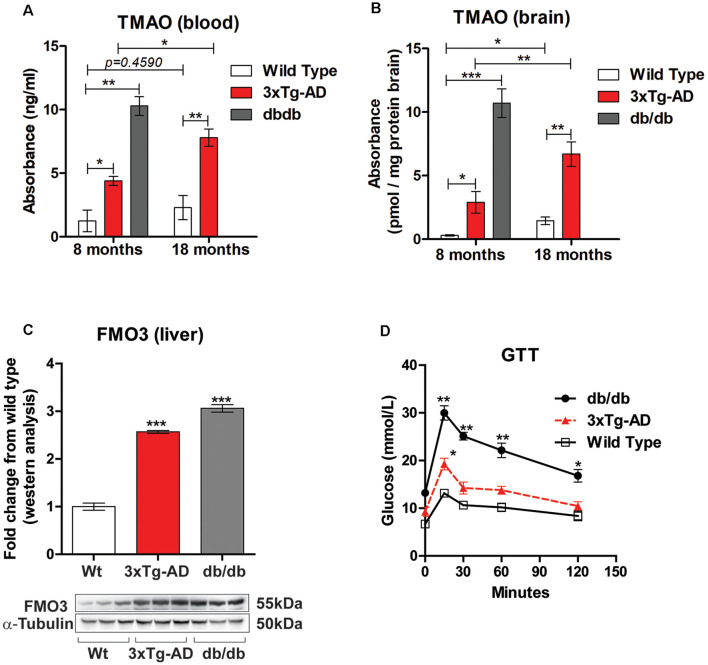
Trimethylamine N-oxide (TMAO) is increased in 3×Tg-AD and db/db mice. TMAO concentrations in the blood **(A)** and brain **(B)** in control, 3×Tg-AD, and db/db mice at 8 months and 18 months. **(C)** Western blot analysis of FMO3 in liver lysates from control, 3×Tg-AD, and db/db mice aged 8 months. α-tubulin was used as an internal housekeeping protein control. **(D)** Eight-month-old Control, 3×Tg-AD, and db/db mice were subjected to glucose tolerance test (GTT) by intraperitoneal glucose administration (2.0 g.kg-1 of body weight). Blood was sampled at 0 (baseline), 30, 60, 90, and 120 min for glucose. Changes in serum glucose levels during the GTT relative to basal glycemic levels. Results are shown as mean ± SEM of three mice per group and *p < 0.05, **p < 0.01 and ***p < 0.001 as compared to different groups.

Alzheimer’s disease is characterized by brain insulin resistance and altered glucose homeostasis and hence also termed as Type 3 diabetes mellitus (de la Monte and Wands, 2008). To determine if TMAO is upregulated in 3×Tg-AD and db/db mice, we measured the levels of TMAO in the blood and in the brain at 8 months (representing early stage) in both the mouse models and compared it to control (wild-type) mice. Similarly, we measured TMAO levels in the blood and brain in 3×Tg-AD mice at 18 months (representing late-stage) and compared it to control mice. We observed that plasma TMAO levels were significantly higher in both 3×Tg-AD (*t*_(4)_ = 3.422, *p* = 0.0267, Figure 1A) and db/db mice (*t*_(4)_ = 8.020, *p* = 0.013, Figure 1A) at 8 months in comparison to control mice. Furthermore, we also observed a statistically significant increase in TMAO levels in the plasma of 18 months 3×Tg-AD when compared to age-matched control mice (*t*_(4)_ = 4.695, *p* = 0.00935, Figure 1A). Next, we measured the TMAO levels in the brain and found significantly elevated levels in 3×Tg-AD (*t*_(4)_ = 3.079, *p* = 0.0370, Figure 1B) and db/db mice (*t*_(4)_ = 9.276, *p* = 0.0008, Figure 1B) at 8 months. Additionally, we found a statistically significant increase in TMAO levels in the brain of 18 months 3×Tg-AD when compared to control mice (*t*_(4)_ = 5.150, *p* = 0.0067, Figure 1B).

Several studies have indicated that the gut microbiota-initiated TMA/FMO3/TMAO pathway regulates metabolic alterations. TMA (trimethylamine) is a metabolite formed by the action of gut microbes on a certain western diet rich in choline, L-carnitine, and phosphatidylcholine. TMA is further metabolized by the hepatic enzyme flavin-containing monooxygenase 3 (FMO3) to produce TMAO. Hence, we sought to investigate the hepatic levels of FMOs in 3×Tg-AD and db/db mice and compared them to control mice. Immunoblot analysis showed a statistically significant increase in FMO3 protein expression in 8-month-old 3×Tg-AD and db/db mice in comparison to control mice (*t*_(4)_ = 19.50, *p* = 0.0001, and (*t*_(4)_ = 18.98, *p* = 0.0001 respectively, Figure 1C). Next, we performed a GTT on these mice to investigate the correlation between increased TMAO to impaired glucose homeostasis. As shown in Figure 1D, the levels of blood glucose in 8-month-old 3×Tg-AD and db/db mice were higher than age-matched control mice at all-time points, however statistically significant results were noted at all-time points only in db/db mice.

### Deficits in LTP Are Noted in 3×Tg-AD, db/db Mice and TMAO Incubated Hippocampal Slices

**Figure 2:**
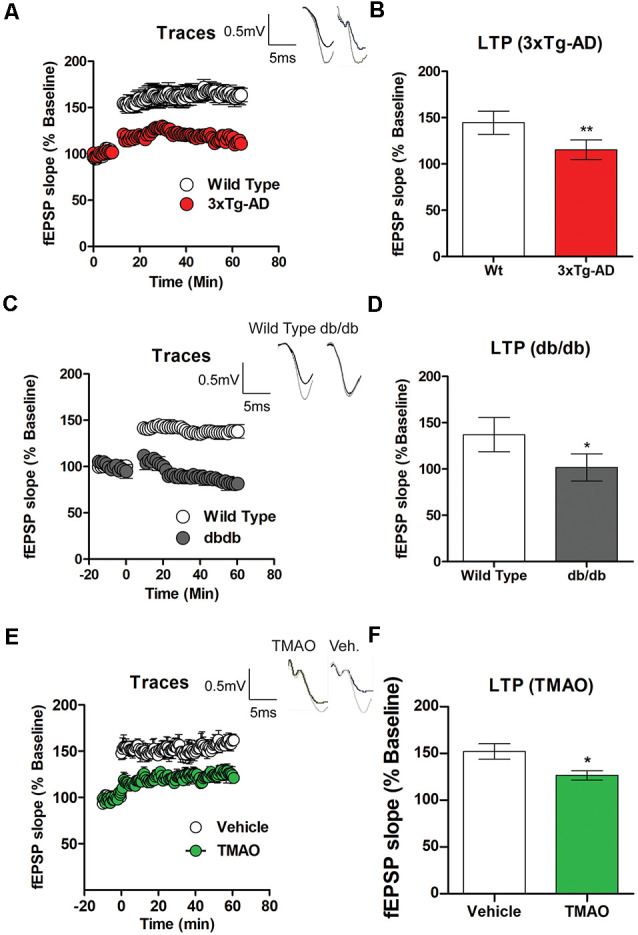
Deficits in long term potentiation (LTP) are noted in 3×Tg-AD and db/db mice. Hippocampal slices from control, 3×Tg-AD, and db/db mice were incubated for 2 h in artificial cerebral spinal fluid (ACSF) before recording. **(A)** LTP graph represents field excitatory postsynaptic potential (fEPSP) slope before and after induction by theta burst stimulation (TBS) in control (wild type) and 3×Tg-AD mice aged 8 months. **(B)** LTP bar graph shows fEPSPs recorded during the period 50–60 min following TBS induction normalized to baseline levels. **(C)** LTP graph comparing control (wild-type) and db/db mice aged 8 months and **(D)** bar graph representing fEPSPs during 50–60 min following TBS induction normalized to baseline levels. Hippocampal slices from control mice were incubated in ACSF with either 0.03% dimethyl sulfoxide (DMSO; vehicle) or TMAO (50 μM) for 4 h before recording. **(E)** LTP graph represents fEPSP slope before and after induction by TBS in vehicle-treated and TMAO treated slices. **(F)** LTP bar graph shows fEPSPs recorded during the period 50–60 min following TBS induction normalized to baseline levels. Bars represent mean ± SEM; *indicates a significant difference between control (wild-type) and db/db or 3×Tg-AD mice and significant difference between vehicle vs. TMAO treated slices, *p < 0.05 for **(A–D)**. Results are shown as mean ± SEM of three to four mice per group and *p < 0.05, **p < 0.01; *n* = 5–6 slices from four mice per group; for **(E,F)** *n* = 5–6 slices from four mice per group.

Cognitive deficits commonly occur in metabolic diseases such as diabetes mellitus patients. Therefore, we investigated LTP- a cellular model of learning and memory in 3×Tg-AD and db/db mice and compared it to wild-type mice. There was a significant reduction in LTP induced by TBS in the Schaeffer collateral pathway in 3×Tg-AD mice in comparison to wild-type mice (*t*_(4)_ = 9, *p* = 0.0022, Figures 2A,B). Similarly, we found a statistically significant deficit in LTP in db/db mice when compared to wild-type mice (*t*_(4)_ = 9, *p* = 0.0053, Figures 2C,D). To determine if TMAO induced deficits in LTP, hippocampal slices incubated with either vehicle or TMAO were used to measure LTP changes. There was a significant reduction in LTP induced by TBS in the Schaeffer collateral pathway in TMAO incubated slices compared to vehicle-treated slices (*t*_(10)_ = 2.514, *p* = 0.03, Figures 2E,F). We hypothesize that alterations in LTP may be in part due to impaired energy regulation causing reduced synaptic activation during LTP induction.

### TMAO Induces Alterations in Basal Synaptic Transmission

**Figure 3:**
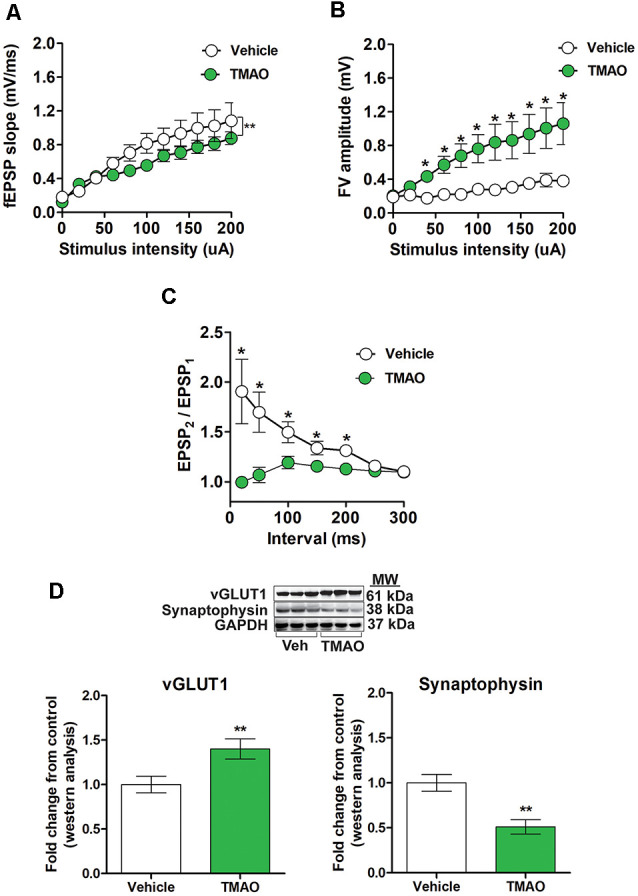
TMAO treated slices exhibits alterations in basal synaptic transmission and paired-pulse facilitation (PPF). For **(A–C)**, hippocampal slices were incubated in ACSF with either 0.03% DMSO (vehicle) or TMAO (50 μM) for 4 h before recording. **(A)** The input-output curve of the fEPSP slope measured at increasing stimulus intensities. **(B)** The input-output curve of FV amplitude measured at increasing stimulus intensities. **(C)** Change in the ratio of second stimulus fEPSP (EPSP2) to the first stimulus fEPSP (EPSP1) slope plotted as a function of interstimulus interval. **(D)** Representative western blot showing vGLUT1 and synaptophysin relative densities normalized to GAPDH in slices treated with either vehicle or TMAO (50 μM); for panels **(A–C)** *n* = 5–6 slices from four mice per group; for **(D)** *n* = 3 mice per group; for panels **(A–C)** planned pairwise comparisons were performed for individual data point analysis for a vehicle vs. TMAO-treated slices. Symbols/bars represent mean ± SEM; *indicates significant difference between vehicle vs. TMAO-treated slices, *p < 0.05, **p < 0.001.

To determine if TMAO alters basal synaptic transmission, hippocampal slices incubated with either vehicle or TMAO were used to measure the fEPSP responses at increasing stimulus intensities. We observed an alteration in fEPSPs over a range of stimulus intensities among two groups (*t*_(10)_ = 4.359, *p* = 0.0014, Figure 3A). fEPSP slopes were reduced in TMAO incubated slices compared to vehicle-treated indicating deficits in baseline glutamatergic synaptic transmission. Next, we sought to investigate whether the deficits in baseline synaptic transmission are to alterations in presynaptic axon recruitment by performing stimulus intensity vs. fiber volley (FV) amplitude on the slices. We observed an increase in FV amplitude with increasing stimulus intensities in TMAO treated slices (*t*_(10)_ = 6.452, *p* = 0.0001, Figure 3B). To further investigate whether the increase in FV amplitude contributed to the presynaptic release probability, we performed PPF, a type of short-term plasticity which depends on residual calcium build-up in the presynaptic terminal was evaluated. A decrease in PPF in TMAO incubated slices at 20, 50, 100, 150, and 200 ms was observed indicating an overall higher presynaptic release probability (*t*_(6)_ = 2.596, *p* = 0.04, Figure 3C). Because alterations in presynaptic proteins involved in glutamate release may contribute to altered PPF, we evaluated the protein levels of vesicular glutamate transporter 1 (vGLUT1), a presynaptic transporter responsible for uptake of glutamate into the synaptic vesicles and synaptophysin, a protein involved in the release of neurotransmitters. We noted a significant increase in vGLUT1 expression (*t*_(4)_ = 4.706, *p* = 0.0093, Figure 3D) and a decrease in synaptophysin density (*t*_(4)_ = 6.929, *p* = 0.0023, Figure 3D) in TMAO treated slices in comparison to vehicle-treated slices suggesting alterations in vesicular glutamate storage and release.

### TMAO Induces a Reduction in Glutamatergic Receptor Subunits

**Figure 4:**
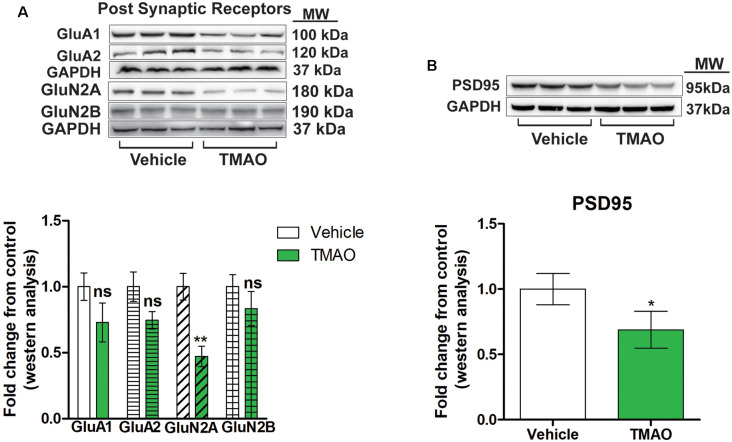
TMAO induces changes in post-synaptic receptor subunits. **(A)** Representative western blot showing GLuA1, GLuA2, GLuN2A, and GLuN2B relative levels normalized to GAPDH in slices treated lysates. **(B)** Representative western blot showing PSD95 relative levels normalized to GAPDH. Symbols/bars represent mean ± SEM; *indicates significant difference between vehicle vs. TMAO treated slices, *p < 0.05, **p < 0.01; *n* = 3 mice per group. ns, non-significant.

A reduction in LTP could be due to alterations in the strength of the signaling during LTP induction or postsynaptic receptor mechanism during LTP maintenance (Lüscher and Malenka, 2012; D’Errico et al., 2009). We examined whether the reduced LTP changes seen in TMAO incubated slices could be due to alterations in post-synaptic glutamatergic receptors expression. The observed alterations in induction and maintenance of LTP may be explained by changes in α-amino-3-hydroxy-5-methyl-4-isoxazolepropionic acid receptor (AMPAR) or N-methyl-D-aspartate receptor (NMDAR) subunit expression levels. Therefore, we measured the protein expression of AMPAR and NMDAR subunits by western blot analysis in protein lysates obtained from TMAO and vehicle-treated hippocampal slices. The AMPAR subunits GluA1 and GluA2 were reduced in TMAO incubated slices (*t*_(4)_ = 1.497, *p* = 0.2008; *t*_(4)_ = 1.989, *p* = 0.1176, respectively, Figure 4A), which may contribute to deficits in LTP along with deficits in basal synaptic transmission. Additionally, there was a reduction in the level of GluN2A (*t*_(4)_ = 6.385, *p* = 0.0031, Figure 4A) and, an NMDAR subunit confirming the reduced receptor activation during LTP induction which is mainly GluN2A dependent (Kariharan et al., 2015; Klann and Sweatt, 2008). However, we observed no significant change in the density of GluN2B (*t*_(4)_ = 1.803, *p* = 0.1457, Figure 4A). Finally, we noted a modest and significant reduction in the level of PSD95 (*t*_(4)_ = 2.920, *p* = 0.0432, Figure 4B) a postsynaptic protein that acts as an anchoring protein. Taken together, our data indicate that reduced levels of glutamatergic receptor subunits may account for the alteration in synaptic plasticity in TMAO treated slices.

### TMAO Induces ER Stress Signaling by Activating the PERK Pathway

**Figure 5:**
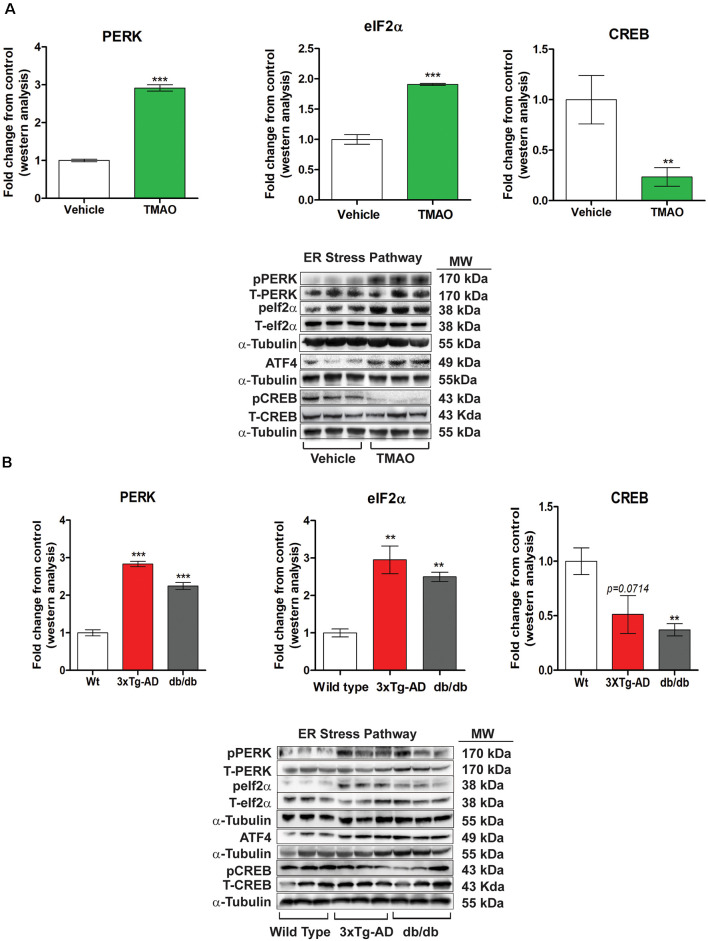
TMAO induces endoplasmic reticulum (ER) stress by activating the PERK pathway of the unfolded protein response (UPR). **(A)** Hippocampal slices were incubated in ACSF with either 0.03% DMSO (vehicle) or TMAO (50 μM) for 4 h and then the lysates were subjected to immunoblotting. Representative western blot showing p-PERK, PERK, p-eIF2α, eIF2α, ATF4, p-CREB, and CREB levels normalized to α-tubulin. **(B)** Western blot analysis of p-PERK, PERK, p-eIF2α, eIF2α, ATF4, p-CREB, and CREB in hippocampal lysates from control, 3×Tg-AD and db/db mice aged 8 months. α-tubulin was used as an internal housekeeping protein control. Results are shown as mean ± SEM of three mice per group. **p < 0.01, ***p < 0.001.

To identify the potential mechanisms by which TMAO induces deficits in synaptic plasticity, we evaluated the changes in key signaling proteins involved in ER stress from the vehicle or TMAO treated slices. Any alterations in endoplasmic reticulum homeostasis can induce the unfolded protein response (UPR). UPR sensory proteins consist of type 1 ER transmembrane protein kinase PERK, IRE1, and ATF6, all three activated by ER stress. TMAO has been shown to bind to PERK and activate the PERK branch of the UPR (Zhuang et al., 2019). Thus, we measured the protein levels of PERK and its downstream signaling effectors to explore the influence of TMAO on UPR. Our results show that TMAO induces an increase in the phosphorylation of PERK in the TMAO treated slices as compared to vehicle-treated slices (*t*_(4)_ = 37.37, *p* = 0.0007, Figure 5A). Furthermore, we observed a statistically significant increase in phosphorylation of eIF2α at Serine 51 in TMAO treated slices (*t*_(4)_ = 5.143, *p* = 0.0068, Figure 5A) which results in increased the protein expression of ATF4 (data not shown). Also, we observed a decrease in phosphorylation of CREB (*t*_(4)_ = 5.143, *p* = 0.0068, Figure 5A) which may also help explain the deficits in synaptic plasticity noted in TMAO treated slices. Similarly, we evaluated the levels of PERK and its downstream signaling in control (wild-type), 3×Tg-AD, and db/db mice aged 8 months. We also observed a statistically significant increase in phosphorylation of PERK (*t*_(4)_ = 16.59, *p* = 0.0001, Figure 5B) in 3×Tg-AD mice and in db/db mice (*t*_(4)_ = 10.01, *p* = 0.0006, Figure 5B) in comparison to control mice. In addition we also observed an increased phosphorylation of eIF2α at Serine 51 in 3×Tg-AD (*t*_(4)_ = 11.54, *p* = 0.0003, Figure 5B) and db/db mice (*t*_(4)_ = 9.74, *p* = 0.004, Figure 5B), and ATF4 expression (data not shown). Similar to TMAO treated slices, we observed a decrease in phosphorylation of CREB with a statistically significant decrease noted in only db/db mice (*t*_(4)_ = 4.635, *p* = 0.0098). However, we saw a minimal decrease in 3×Tg-AD mice (*t*_(4)_ = 2.284, *p* = 0.0714). These results indicate that TMAO mediated deficits in synaptic plasticity may be attributed to the induction of ER stress through the PERK-eIF2α signaling pathway and its downstream signaling axis.

### TMAO Induces an Increase in Oxidative Stress Markers

**Figure 6:**
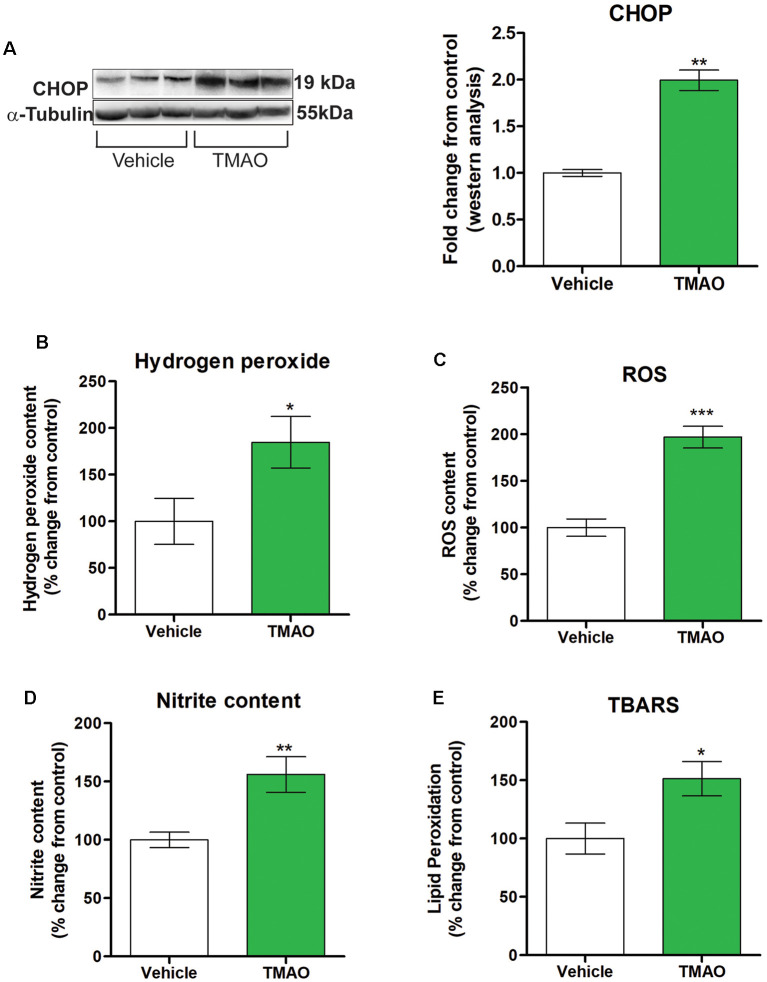
TMAO induces oxidative stress. Hippocampal slices were incubated in ACSF with either 0.03% DMSO (vehicle) or TMAO (50 μM) for 4 h and the lysates were analyzed for CCAAT-enhance-binding protein homologous protein (CHOP) protein expression and oxidative markers. **(A)** Representative western blot showing CHOP levels normalized to α-tubulin. **(B)** Hydrogen peroxide generation in the lysates was measured fluorometrically at Ex/Em 535/587 nm. **(C)** ROS generation was assayed using DCF dye and measured with a spectrophotometer. **(D)** Nitrite content was measured using Griess reagent and the azo product formed was measured spectrophotometrically at 545 nm. **(E)** Lipid peroxidation was estimated by measuring the malondialdehyde (MDA) content in the form of thiobarbituric acid reactive substances (TBARS method) spectrophotometrically. For **(A)** symbols/bars represent mean ± SEM; *indicates a significant difference between vehicle vs. TMAO treated slices, *n* = 3 mice per group, *p < 0.05. For **(B–E)** results are expressed as (%) change as compared to the control, Mean ± SEM; *p < 0.05, **p < 0.01, ***p < 0.001.

Disruption of normal cellular homeostasis by redox signaling has been shown to play a significant role in the pathogenesis of cognitive impairment noted in various metabolic and neurodegenerative disorders (Zhuang et al., 2019). Downstream of ATF4 is CCAAT-enhance-binding protein homologous protein (CHOP), which is induced by ER stress and mediates inflammation and apoptosis. We investigated the expression levels of CHOP in TMAO and vehicle-treated slices and found increased protein expression of CHOP in TMAO treated slices in comparison to vehicle-treated slices (*t*_(4)_ = 5.143, *p* = 0.0010, Figure 6A). ROS is classified either into oxygen radicals (superoxide, hydroxyl, peroxyl, and alkoxyl) or the nonradicals (hypochlorous acid, ozone, singlet oxygen, and hydrogen peroxide). Hence, we investigated the effect of TMAO on various redox markers in TMAO incubated slices in comparison to vehicle-treated slices. We noted a statistically significant increase in hydrogen peroxide levels (*t*_(10)_ = 2.292, *p* = 0.0224, Figure 6B) and ROS generation (*t*_(10)_ = 6.492, *p* = 0.0001, Figure 6C) in TMAO treated slices in comparison to vehicle-treated group. The generation of reactive oxygen species (ROS) induces irreversible oxidation of lipids and protein. Therefore, we determined the effect of TMAO on lipid peroxidation and nitrite content in TMAO and vehicle-treated slices. We found a statistically significant increase in nitrite (*t*_(10)_ = 3.376, *p* = 0.0071, Figure 6D) and lipid peroxidation (*t*_(10)_ = 2.587, *p* = 0.0271, Figure 6E) levels in TMAO treated slices.

## Discussion

Studies in rodent models and clinical studies have linked TMAO with Alzheimer’s disease, diabetes mellitus, and cardiovascular diseases (Wang et al., 2011; Dambrova et al., 2016; DiNicolantonio et al., 2019; Zhuang et al., 2019). Our findings in Figure 1 support the knowledge from clinical findings for understanding the association of TMAO with diabetes and AD (Dambrova et al., 2016; DiNicolantonio et al., 2019). We observed approximately a 10-fold increase in circulating TMAO in diabetic (db/db) mice when compared to non-diabetic mice. Diabetes and Alzheimer’s disease share commonalities, including hyperglycemia, hyperinsulinemia, and memory impairment. Recent evidence has linked microbiome dysbiosis to be associated with neurological conditions including neuroinflammation and AD (Jiang et al., 2017; Cerovic et al., 2019; Askarova et al., 2020). In transgenic rodent models of AD, gut dysbiosis had positive correlations with AD pathology including cerebral amyloid-beta depositions (Cattaneo et al., 2017). Higher concentrations of TMAO in circulation and CSF have been observed in diabetic patients as well as patients with MCI and Alzheimer’s disease (AD) dementia (Vogt et al., 2018). TMAO is elevated in individuals with diabetes (Abubacker et al., 2001; Dambrova et al., 2016) and has been shown to promote insulin resistance in mice fed a high-fat diet (Gao et al., 2014). Given that diabetes mellitus and insulin resistance are risk factors for developing AD (Adler et al., 2014), elevated TMAO in the CNS may exacerbate central insulin resistance and AD pathogenesis. Recent findings indicate that elevated TMAO in circulation, due to dietary choline supplement, promotes brain aging and cognitive impairment (Li et al., 2018). However, many of these reports focus upon correlating levels of memory impairment with serum levels and or cerebrospinal fluid levels of TMAO. In the present study, we provide biochemical evidence revealing that TMAO from serum and brain lysate is higher in db/db leptin-deficient mice and 3×Tg-AD mice. In multiple clinical studies, systemic levels of TMAO were observed to strongly associate with type 2 diabetes mellitus (Jia et al., 2019; Zhuang et al., 2019). Also, circulating TMAO levels were associated with obesity traits in the different inbred strains represented in the Hybrid Mouse Diversity Panel (Lusis et al., 2016). Further, antisense oligonucleotide-mediated knockdown, or genetic deletion of the TMAO-producing enzyme, flavin-containing monooxygenase 3 (FMO3), offers protection against hyperglycemia, hepatic insulin resistance, and obesity in mice (Schugar et al., 2017). We observed a significant increase in blood glucose levels in 3×Tg-AD and db/db mice when compared to age-matched wild-type mice. These hyperglycemic levels help explain the elevated hepatic FMO3 levels and the subsequent elevated TMAO circulating levels in 3×Tg-AD and db/db mice when compared to wild-type mice. Besides, we also observed that TMAO is significantly elevated in aged diabetic and AD mice, and exposure to TMAO leads to enhanced ER stress and impaired synaptic plasticity.

The synthesis, folding, and transport of all plasma membrane channels and receptors essential for synaptic function are coordinated *via* endoplasmic reticulum. Several acute and chronic disease conditions impair ER function leading to ER stress, including Alzheimer’s disease and diabetes. Recent work by Chen et al. (2019) identified that the endoplasmic reticulum stress kinase PERK acts as a receptor for TMAO, activates PERK mediated phosphorylation of eIFα, and promotes hyperglycemia. Forebrain PERK knock-out mice demonstrated that stimulation of early- and late-phase LTP (E-LTP and L-LTP, respectively) is associated with decreased eIF2α phosphorylation (Trinh et al., 2014). Further work by Sharma et al. (2018) showed that reduction of PERK expression and activity *via* viral vector delivery in the CA1 region of the hippocampus enhances hippocampal function and improves hippocampal-dependent learning in middle-aged male rodents. Consistent with these findings, heterozygous mutant eIF2α knock-in mice display a decreased threshold for the induction of LTP (Costa-Mattioli et al., 2007). Furthermore, hippocampal infusion with Sal003, an inhibitor of eIF2α dephosphorylation (Boyce et al., 2005), prevented the induction of both LTP and long-term memory (Costa-Mattioli et al., 2007).

We observed a significant increase in PERK activation and eIF2α activation (phosphorylation) in 3x-Tg-AD mice, db/db mice, and slices incubated with TMAO. In Figure 2 we observed significantly reduced LTP in 8-month-old 3×Tg-AD and db/db mice. We have previously reported that rodent models of diabetes displayed significant behavioral deficits and reduced LTP (Shonesy et al., 2012; Kariharan et al., 2015). In response to stress, neurons can alter their molecular and physiological characteristics. Synaptic plasticity is such a response by the neurons to change the strengthening or weakening of synaptic connections between neurons (Bliss and Collingridge, 1993). This activity-dependent, changes in synaptic strength are triggered by *de novo* protein synthesis (Klann and Sweatt, 2008). These findings indicate that protein synthesis can be triggered locally at activated synapses and is required for persistent, activity-dependent forms of synaptic plasticity, which in turn is thought to be essential for memory formation. Notably, in multiple species ATF4 and its homologs act as repressors of cAMP-responsive element-binding protein (CREB)-mediated gene expression, which is known to be required for long-lasting changes in synaptic plasticity and long term memory (Chen et al., 2003; Bartsch et al., 1995; Abel et al., 1998). Thus, eIF2α phosphorylation controls both general and gene-specific translation that regulates CREB-mediated transcription, two distinct processes that are required for long-lasting synaptic plasticity and long-term memory formation. Our findings are consistent with this concept that we observed increased levels of ATF4 and reduced CREB levels. Further, that ATF4 also regulates CHOP which results in enhancing inflammation.

In the current study, we observed reduced LTP in both 3×Tg-AD and db/db mice. Besides, both models also displayed reduced AMPAR and NMDAR subunits. To better understand the direct impact of TMAO on LTP we incubated brain slices treated with TMAO and observed a modest reduction in LTP ≈ 26%. We are the first to report the impact of TMAO on LTP. To help explain these findings reduced levels of AMPAR and NMDAR subunits may be responsible for the impairments in LTP induction and preservation. The reduction in glutamatergic receptor subunits including GluA1 and GluN2A is related to a reduction in LTP and basal synaptic transmission. An increase in presynaptic release induced by TMAO is probably endoplasmic reticulum oxidoreductin-1α (ero1-α) dependent, which might help explain the increased basal synaptic transmission.

**Figure 7:**
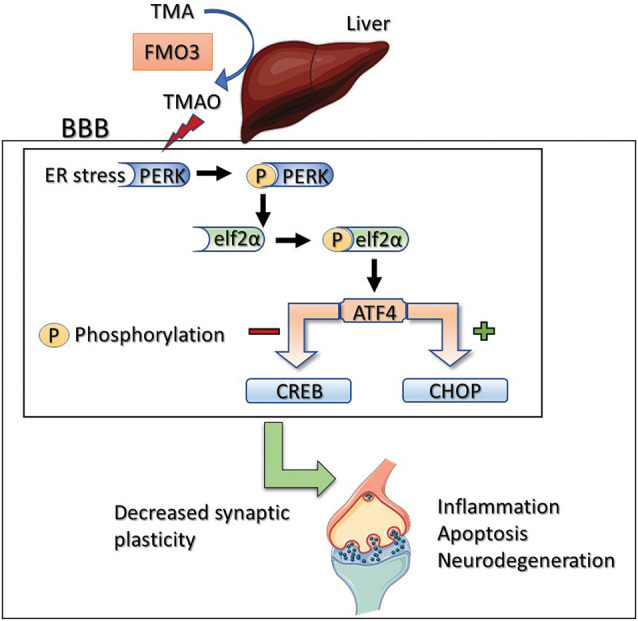
Proposed mechanism of TMAO induced synaptic plasticity deficits. Activation of PERK by TMAO leads to phosphorylation of eIF2α which can directly inhibit general protein synthesis (mRNA translation) or activate ATF4. ATF4 negatively regulates synaptic plasticity and memory by acting as a repressor of CRE-dependent transcription (CREB). Alternatively, ATF4 translocates to the nucleus and increases the expression of CHOP which promotes reactive oxygen species formation, inflammation, and apoptosis.

vGLUT is responsible for the uptake of glutamate into synaptic vesicles at the presynaptic terminals and thus is related to the number and fill state of presynaptic vesicles and release probability in hippocampal neurons (Fremeau et al., 2001). In the current study, the increase in VGLUT1 expression suggests an increase in the availability of presynaptic vesicles and the presynaptic release probability in TMAO incubated slices. Recent work by Terashima et al. (2019) demonstrated that removal of GluA1 in neurons resulted in complete loss of hippocampal LTP and a reduction in synaptic transmission. Further that the reduction in our studies of GluA1 may be due to the increase in presynaptic release (vGLUT1) mediated glutamate exposure. Further studies using PERK or ero1-α inhibitors will be valuable in elucidating mechanisms by which TMAO alters synaptic transmission and LTP. Based on our findings, we suggest that TMAO influences the hippocampal synaptic transmission by altering the glutamatergic signaling pathway. In conclusion, our results suggest that the elevated FMO3 in the liver induces an increase in TMAO which induces ER stress in the brain. The protein misfolding associated with elevated TMAO results in impaired synaptic transmission and LTP. Previous studies have demonstrated that TMAO induces inflammation and oxidative stress (Seldin et al., 2016). Similarly, our results indicate that TMAO induces oxidative stress by increasing ROS, hydrogen peroxide levels, lipid peroxidation, and nitrite content in hippocampal slices. Furthermore, oxidative stress is strongly associated with cognitive impairment, leading to reduced life quality and expectancy. Hence, the results of our study indicate a strong association between TMAO and cognitive deficits (Figure 7). Indeed, the presence and elevation of TMAO in CSF from Alzheimer’s and diabetic patients indicate an essential cross-talk between gut-liver and brain. Development of novel therapeutics targeting TMAO, especially the enzyme FMO3 through specific inhibition could help in attenuating the cognitive deficits seen in AD and other diseases with cognitive impairment.

## Data Availability Statement

All datasets presented in this study are included in the article.

## Ethics Statement

The animal study was reviewed and approved by Auburn University Animal Care and Use Committee.

## Author Contributions

MG, IS and RA conceived and designed the experiments. MG, PP, IS, JB, SR, RR, SB, MD and RA performed the experiments and/or analyzed the data. MG wrote the manuscript with support from RA and VS. All authors contributed to the article and approved the submitted version.

## Conflict of Interest

The authors declare that the research was conducted in the absence of any commercial or financial relationships that could be construed as a potential conflict of interest.
